# Pain prevalence and pain management in children and adolescents in an italian third level pediatric hospital: a cross-sectional study

**DOI:** 10.1186/s13052-023-01439-2

**Published:** 2023-03-29

**Authors:** Giuliano Marchetti, Alessandro Vittori, Marco Cascella, Ilaria Mascilini, Simone Piga, Emiliano Petrucci, Aurora Castellano, Roberta Caruso, Elisa Francia, Francesca Stocchi, Franco Marinangeli, Alessandro Inserra, Sergio Giuseppe Picardo

**Affiliations:** 1 Department of Anesthesia and Critical care, ARCO Roma, Ospedale Pediatrico Bambino Gesù IRCCS, Piazza S. Onofrio 4, 00165 Rome, Italy; 2 Department of Anesthesia and Critical Care, Istituto Nazionale Tumori-IRCCS, Fondazione Pascale, Naples, Italy; 3 Unit of Clinical Epidemiology, Ospedale Pediatrico Bambino Gesù IRCCS, Rome, Italy; 4 Department of Anesthesia and Intensive Care Unit, San Salvatore Academic Hospital of L’Aquila, L’Aquila, Italy; 5 Division of Oncohematology, Ospedale Pediatrico Bambino Gesù IRCCS, Rome, Italy; 6 Department of Anesthesiology, Intensive Care and Pain Treatment, University of L’Aquila, L’Aquila, Italy; 7 Surgical Department, General and Thoracic Unit, Ospedale Pediatrico Bambino Gesù IRCCS, Rome, Italy

**Keywords:** Pain, Pain management, Pain management index, Analgesic score, Children, Pediatric pain, Opioid, Anesthesia, Cancer

## Abstract

**Background:**

In 2016, we performed a one-day investigation to analyze the prevalence of pain, pain intensity, and pain therapy in the Departments of Surgery and Onco-Hematology of the Ospedale Pediatrico Bambino Gesù. To improve the knowledge gap highlighted in the previous study, refresher courses and even personalized audits have been carried out during these years. The purpose of this study is to evaluate if, after 5 years, there have been improvements in the management of pain.

**Methods:**

The study was conducted on 25 January 2020. Pain assessment, pain therapies, pain prevalence and intensity in the preceding 24 h and during the recovery period were recorded. Pain outcomes were compared with previous audit results.

**Results:**

Out of the 63 children with at least one documented pain assessment (starting from 100 eligible), 35 (55.4%) experienced pain: 32 children (50.7%) experienced moderate /severe pain while 3 patients (4%) felt mild pain. In the preceding 24 h, 20 patients (31.7%) reported moderate/severe pain while 10 (16%) reported moderate or severe pain during the interview. The average value of the Pain Management Index (PMI) was − 1.3 ± 0.9 with a minimum of -3 and a maximum of 0. 28 patients (87%) undergoing analgesic therapy for moderate/severe pain had a PMI of less than 0 (undertreated pain), while 3 patients (13%) scored value of 0 or higher (adequate pain therapy), 4 patients (12.5%) received multimodal analgesia with opioids and 2 patients (6%) opioids alone. Time-based therapy was prescribed to 20 patients (62.5%), intermittent therapy was prescribed to 7 patients (22%) and 5 patients (15.5%) did not receive any therapy. The prevalence of pain was higher during hospitalization and 24 h before the interview, while at the time of the interview, the proportion was the same. In this audit, the daily prescription modality of the therapy had some improvements (time-based: 62.5% vs. 44%; intermittent: 22%vs 25%; no therapy: 15.5% vs. 31%).

**Conclusion:**

Pain management in hospitalized children constantly requires special daily attention from health professionals aimed at mitigating the components of intractable pain and resolving those of treatable pain.

**Trial registration:**

: This study is registered with ClinicalTrials.gov, number (NCT04209764), registered 24 December 2019, https://clinicaltrials.gov/ct2/show/NCT04209764?term=NCT04209764&draw=2&rank=1.

**Supplementary Information:**

The online version contains supplementary material available at 10.1186/s13052-023-01439-2.

## Background

Pain control has been universally recognized as a human right for years and correct pain assessment is now one of the standards for the accreditation of healthcare institutions [1–3]. Unfortunately, pain evaluation and treatment are still important health issues in hospitalized patients [4]. Proper pain management can reduce the incidence of complications, reduce days of hospitalization, achieve faster discharges, and decrease the use of hospital resources [4, 5]. Moreover, inadequate pain management can lead to persistent or chronic pain, alterations of the nociception as well as emotional and psychological complications [4, 6, 7]. In fact, pain can have negative effects on the physical and mental conditions of hospitalized patients, worsening the quality of life and increasing costs [8]. Notably, several investigations demonstrated the occurrence of pain, even of moderate or severe degree, between 20% and 50% of hospitalized children [9–20]; this is often associated with inadequate therapy [20]. In fact, despite the ability to accurately assess pain and the considerable technical resources available, the prevalence of pain in hospitalized children is still high with more than onehalf caused by insufficient pain management [21–23].

In 2016, with a one-day investigation, we analyzed the prevalence of pain, pain intensity, and pain therapy in the Department of Surgery (DS) and the Department of Onco-Hematology and Cell and Genetic Therapy (DO) of the largest pediatric hospital in Italy, Ospedale Pediatrico Bambino Gesù IRCCS, which joined the project “Towards a Hospital without pain” some years ago. Our study showed suboptimal pain management and also suggested the need to take further initiatives to improve pain management in the wards [20].

To improve the knowledge gap highlighted in the previous study, refresher courses and even personalized audits have been carried out during these years. The purpose of this study is to evaluate if, after 5 years, there have been improvements in the management of pain.

The primary objective of this study is to describe the prevalence of pain in children in ordinary and day hospitalization in the DS and the DO. Secondly, the study aims (1) to assess the proportion of patients with pain who are receiving painkilling treatment; (2) to describe the type of analgesic drugs used and the dosage administered; (3) to evaluate the adequacy of the analgesic therapies administered, with reference to the World Health Organization (WHO) guidelines (WHO Analgesic Scale); (4) to evaluate the Pain Management Index (PMI) [20, 24–26].

## Methods

In this cross-sectional and monocentric study, all patients in ordinary or day hospitalization in the Department of Surgery (DS) and the Department of Onco-Hematology and Cell and Genetic Therapy (DO) were included; no study-specific exclusion criteria were applied. If patients with language barriers were present in the wards, the protocol also envisaged their enrollment since datareport a sub-optimal pain therapy in this patient category [27, 28].

The study was approved by the Ethics Committee of the Ospedale Pediatrico Bambino Gesù IRCCS (1744_OPBG_2019) and it was registered with ClinicalTrials.gov, number (NCT04209764). The study was conducted on 25th January 2020.

Pain prevalence was calculated as the proportion of patients reporting pain sensation out of the total number of patients included in the investigation.

Pain was assessed according to the Hospital Pain Assessment Protocol previously described [20]: during the hospitalization period, all patients got a pain assessment. In particular surgical patients were assessed upon entering the ward for hospitalization and returning after surgery, and then 3 times a day and each time the patient or parents reported pain. For onco-hematological patients the pain assessment was made at the entrance and then at least four times a day (every 6 h).

Pain was assessed during usual care. The individual pain intensity scores obtained were converted to a common four-level metric (none, mild, moderate, and severe pain). For the Verbal Descriptive Scale, the scores were converted into four levels whereby no pain was converted to none; a little pain to mild; medium pain to moderate; and a lot of pain to severe. For the Numerical Rating Scale (NRS), the Visual Analogic Scale (VAS), and the Face, Legs, Arms, Cry and Consolability Scale (FLACC) scores were also converted as follows: 0 = no pain, 1 to 3 = mild pain, 4 to 6 = moderate pain and > 6 = severe pain [23].

The relevant data for the study were obtained from the patient’s health records and through the administration of a questionnaire to hospitalized patients or their parents.

For the purposes of the study, the following information will be collected:

- Gender;
- Date of birth;
- Weight;
- Date of admission;
- Reason for admission;
- Ward of hospitalization and department;
- Presence of language barriers;
- Cognitive impairment of pain before admission;
- Presence of pain detected at different times: before hospitalization, during hospitalization, at the time of the interview and in the 24 h prior to the interview;
- Pain intensity detected at different times (before hospitalization, during hospitalization, at the time of the interview and in the 24 h prior to the interview) and defined as mild or moderate-severe [23];
- Administration of analgesic drugs;
- Type of analgesia;
- Dosage of analgesic drugs;
- Frequency of administration per hour or in continuous infusion over 24 h;
- Value of the PMI (Pain Management Index);
- Pain assessment;
- Frequency of pain assessment;
- Changes to analgesic therapy, if moderate pain;
- Efficacy of the analgesic drug administered;
- Adequacy of the administered analgesic drug defined according to WHO guidelines;
- Adequacy of information on pain relief at discharge.

To reduce the effect of potential distortions during the data collection phase, all questionnaires were administered in a standardized manner by trained personnel, not directly involved in the care of the subjects of the study and not aware of the start date of the survey. The survey was carried out during a specific time interval (from 8:00 AM to 05:00 PM) on a single day, in order to obtain a picture of the pain prevalence in a common working day in the Departments concerned. The survey was conducted by 4 investigators consisting of 4 pediatric anesthesiologists with specific pain management training, not involved in the subjects’ care, with the task of administering informed consent, (provided for parents and adolescents) and a data collection questionnaire for hospitalized patients or their parents. In the case of patients and families with language barriers, the intervention of cultural mediators was envisaged. The questionnaire was administered only after the acquisition of informed consent.

Interviewers used a data collection form on which the following parameters were recorded:

1. Demographic: age, sex, weight, presence of language barriers or cognitive deficits of pain before hospitalization and during hospitalization.
2. Pain: pain assessment by FLACC scale for children up to 4 years and by NRS or VAS scales for children up to 18 years. The presence, intensity and characteristics of pain was observed at the time of the interview and in the previous 24 h.
3. Analgesia: administration of analgesic drugs, type of analgesia and frequency of drug administration, modifications to therapy if moderate pain, adequacy of the analgesic drug administered according to the WHO guidelines, PMI which derives from the relationship between the Pain Score (derived from pain assessment) and Analgesic Score (derived from the type of drug used in relation to the analgesic scale WHO: no analgesic 0 points; WHO I: 1 point; WHO II: 2 points; WHO III: 3 points). Finally, using a scale from 1 to 10 points (poor = 0–2; sufficient = 3–4; good = 5–6, very good = 7–8; excellent 9–10) the patients or their relatives were able to evaluate the efficacy and the adequacy of the information received at discharge on pain relief therapy [24, 29].

### Statistical analysis

Categorical variables were summarized using absolute frequencies and percentages, and continuous variables by mean and standard deviation. To determine statistical differences between groups, we used the Chi-square test or Fischer’s exact test for categorical variables when appropriate and the t test for continuous variables.

Statistical analyses were carried out using the Stata program, version 17 (2017, Stata statistical.

software: StataCorp, College Station, TX).

## Results

### Demographics

**Fig. 1:**
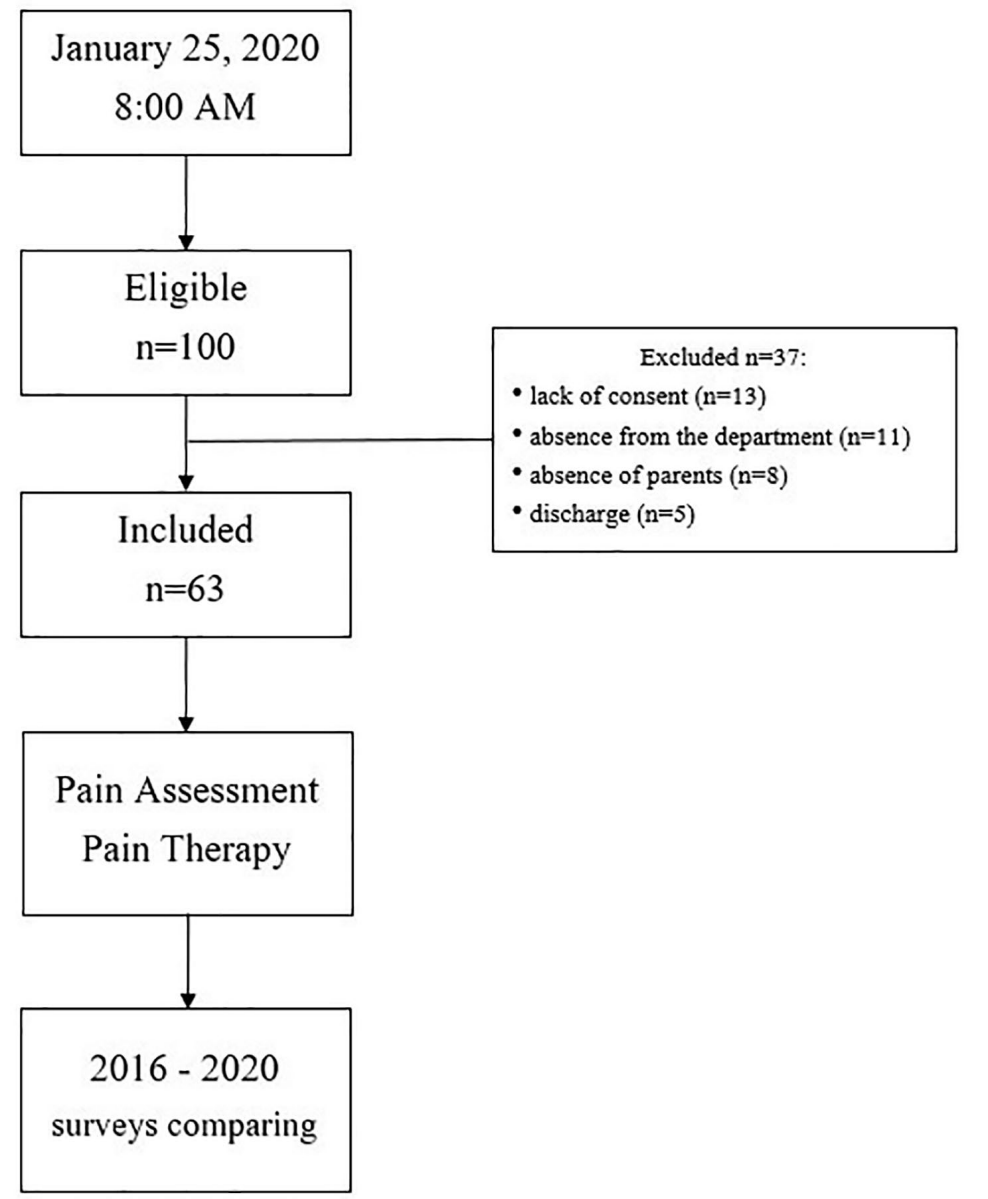
Study flow chart

At 8:00 AM of the day of the survey, 100 patients were identified to be interviewed. Out of the 100 selected, 37 patients (37%) were excluded for lack of consent (n = 13), absence from the department (n = 11), absence of parents (n = 8), discharge (n = 5). Finally, 63 (63%) children (36 male and 27 female, aged 8.3 ± 5.9, min 3.6 months, max 18,0 years) participated in the study (Fig. 1). Out of them, 21 (33%) were admitted to the Department of Onco-Hematology (DO) and 42 (67%) to the Department of Surgery (DS) (Table 1).

**Table 1:** Demographics

Department	Oncohematology n.21 Surgery n.42
Age	8.2 ± 5.7 (min 3.6 months, max 18,0 years)
Gender	Male n.36 Female n.27
Total	n.63

### Pain assesment

The VAS was the most frequently used tool to assess pain (32 patients; 51%), followed by the FLACC Scale (26 patients; 41%) and finally the NRS (5 patients; 8%).

### Pain prevalence

Out of the 63 children with at least one documented pain assessment, 35 (55.4%) experienced some pain. Regarding moderate and severe pain, 32 children (50.7%) experienced moderate /severe pain (M/SP) while 3 patients (4%) experienced mild pain. In the preceding 24 h, 20 patients (31,7%) reported M/SP while 10 (16%) reported M/SP during the interview (Table 2).

**Table 2:** Moderate/Severe Pain Prevalence

Patients (n)	63
During hospitalization	50.7%
24 h before interview	31.7%
At time of interview	16%

### Pain therapy

**Fig. 2:**
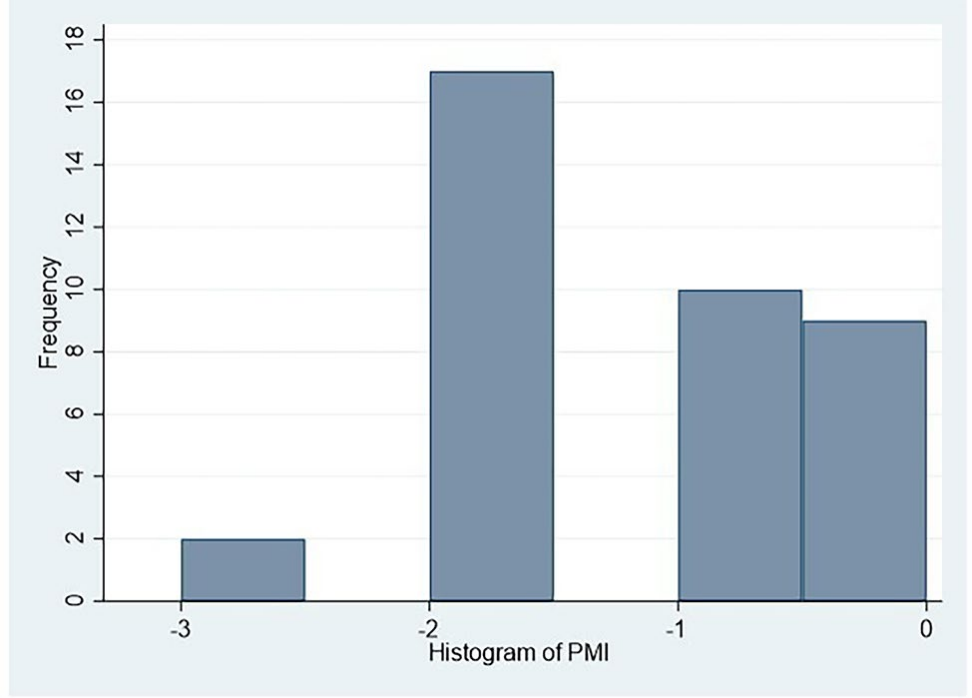
Distribution of PMI Legend: Minimum: -3; 1st Quartile: -2; Median: -2; 3rd Quartile: -1; Maximum: 0.0; Mean − 1.3; Standard deviation: 0.9

For all patients with moderate/severe pain during hospitalization, the PMI was calculated in order to assess the appropriateness of analgesic therapy (Fig. 2).

The average value of PMI was − 1.3 ± 0.9 with a minimum of -3 and a maximum of 0. 87% (28 pts) of children with analgesic therapy for M/S P had a PMI of less than 0 (undertreated pain), while 13% (3 pts) had a value of 0 or > 0 (adequate pain therapy) and only 4 patients (12.5%) received a multimodal analgesia with opioids and 2 patients (6%) opioids alone. Time based therapy was prescribed to 20 patients (62.5%), intermittent therapy was prescribed to 7 patients (22%) and 5 patients (15.5%) did not receive any therapy (Table 3).

**Table 3:** Therapy for Moderate/Severe Pain

Patients with M/S P	32
Mean value PMI	-1.3
Standard Deviation PMI	± 0,9
Minimum PMI	-3
Maximum PMI	0
PMI < 0 (undertreated Pain)	87%
PMI = or > 0	13%
Time Based therapy	62.5%
Intermittent Therapy	22%
No Therapy	15.5%
Multimodal Analgesia	12.5%
Opioids alone	6%

Patients and/or parents rated the efficacy of therapies by a mean value of 6.5 ± 2 with a minimum of 0 and a maximum of 8. 22 (69%) patients and/or parents were informed about pain and pain therapy, while 10 (31%) did not receive any information. The information was considered very good in 36% (8722); good in 32% of cases (7/22); sufficient in 23% (5/22) and poor in 9% (2/22).

All patients with pain therapy received adequate information on home therapy.

**Table 4:** Differences for M/SP prevalence and pain therapy for M/SP between the two departments

	Oncohematology Department	Surgery Department	p-value
Patients n	21	42	
Patients with M/S P	9	23	
M/SP During hospitalization	43% (9/21)	55% (23/42)	0.373
M/SP 24 h before interview	14% (3/21)	40% (17/42)	**0.046**
At time of interview	5% (1/21))	21% (9/42)	0.144
Mean value PMI	-1	-2	**< 0.001**
Standard Deviation PMI	± 0.7	± 0.7	
Minimum PMI	-2	-3	
Maximum PMI	0	0	
PMI < 0 (undertreated Pain)	78% (7/9)	91% (21/23)	0.557
PMI = or > 0	22% (2/9)	9% (2/23)	
Time Based therapy	67% (6/9)	61% (14/23)	1.000
Intermittent Therapy	33% (3/9)	17% (4/23)	0.370
No Therapy	0	22% (5/23	0.288
Multimodal Analgesia	11% (1/9)	13% (3/23)	1.000
Opioids alone	22% (2/9)	0	0.073

**Legend:** The number of patients for each variable taken into consideration are indicated in brackets.

Patients on DS experienced more pain than those on DO (Table 4) during the entire hospitalization (55% vs. 43%), in the 24 h before (40% vs. 14%; p value = 0.046) and during the interview (21% vs. 5%). Furthermore, patients with M/SP admitted tothe DS had worse pain therapy than those admitted to DO. In fact the difference in the mean values of the PMI between the two departments is statistically significant ( p < 0.001) and 91% of surgical patients with M/SP presented a PMI below 0 (undertreated pain) compared to 78% of patients admitted to the DO (Table 4).

### Comparison with previous audit

The comparison between the two audits about the M/SP and the relative administered therapy are shown respectively in Tables 5 and 6. Table 5 shows that the prevalence of pain was higher both during hospitalization and 24 h before the interview in the present audit, while at time of interview the proportion was the same.

**Table 5:** Moderate/severe Pain Prevalence Comparison between the two audits

Audit YEAR	2016	2020	*p*-value
Patients (n)	75	63	
During hospitalization	43%(32/75)	51%(32/63)	0.340
24 h before interview	29%(22/75)	32%(20/63)	0.759
At time of interview	16%(12/75)	16%(10/63)	0.984

**Table 6:** Therapy for Moderate/Severe Pain Comparison between the two audits

Audit YEAR	2016	2020	p-value
Patients n	75	63	
Patients with M/S P	32 (43%)	32 (55%)	0.131
Mean value PMI	− 0.8	-1.3	**0.011**
Standard Deviation PMI	± 1.3	± 0,9	
Minimum PMI	− 3	-3	
Maximum PMI	+ 2	0	
PMI < 0 (undertreated Pain)	60%(45/75)	87%(55/63)	**< 0.001**
PMI ≥ 0	40%(30/75)	13%(8/63)	
Time Based therapy	44%(33/75)	63%(40/63)	**0.038**
Intermittent Therapy	25%(40/75)	22%(14/63)	
No Therapy	31%(23/75)	15%(9/63)	
Multimodal Analgesia	23%(17/75)	13%(8/63)	0.130

Table 6 shows conflicting results: if on the one hand the daily prescription modality of the therapy had improvements (time based: 62.5% vs. 44% with p = 0.038); intermittent: 22%vs 25%; no therapy: 15.5% vs. 31%), on the other, the quality of the prescription did not help much to relieve pain (PMI < 0: 87% vs. 60% with p < 0.001; mean values of the PMI between the two departments is statistically significant (p < 0.001); PMI ≥ 0 in 23.4% vs. 40%; multimodal analgesia 12.5% vs. 23%).

## Discussion

Our investigation highlighted results that are partly disappointing and partly contradictory. In fact, the prevalence of M/SP was higher than in the previous audit, both during hospitalization and in the 24 h prior to the day set for the investigation. All this despite the hospital training initiatives addressed to physicians and nurses, such as the update of analgesic protocols and the organization of biannual continuing medical education (CME) courses on pain therapy. This is all the more true in light of the fact that there are periodic checks on the knowledge of the protocols by the healthcare workers, and these protocols involve a systematic measurement of pain.

On the other hand, the daily prescription of pain therapy improved markedly in both hourly and as-needed indications. In addition, fewer children were prescribed no pain therapy than in the 2016 survey. However, this last positive data is accompanied by poor quality of the analgesic therapy even compared to the previous audit. In fact, the therapy administered resulted in a statistically significant under treatment of pain so that it was unable to alleviate much of the M/SP complained of by the patients.

During hospitalization and in the 24 h before and during the interview, the prevalence of pain was lower in the DO than in the DS. This result probably reflects not only the difference in the characteristics of pain in the two departments (chronic rather than acute), but also a therapy which, although inadequate, was found to be better than that administered in the DS. In fact in the DO slightly better values in PMI, in the administration of hourly therapy and in the use of opioids were observed, and all patients with M/SP had a prescribed pain therapy. On the other hand, the use of as-needed therapy and multimodal analgesia was worse in DO.

The determining factors of pain management practices are: (1) Factors related to health care professionals; (2) Organizational factors; (3) Factors relating to the parents; (4) Factors relating to the child [30]. Among the factors relating to the staff, this paper suggests the existence of a knowledge gap about pain therapy for M/SP concerning multimodal analgesia and opioids [31]. Unfortunately, in Italy, opioid epidemics are discounted, even if the situation is quite the opposite [32]. In fact, the opioid epidemics originated at the time of the previous audit in a completely different context from the Italian one. A health organization based on an insurance system that fostered the prescription of opioids rather than the use of other drugs or other more expensive analgesic techniques has led to excessive use, or rather misuse and abuse, of opioids in North America [33].

In Italy the context is completely different, so much so that a law (38/2010) was necessary to encourage the use of opioids and eliminate the excess of bureaucracy that weighed on the prescription of opioids [31, 34]. Opioid epidemics shifted attention to a problem that did not concern us, blocking a virtuous process that had seen an increase in the prescription of opioids in the right contexts. This has resulted in a retreat in the use and prescription of opioids, with unjustified alerts [35–37].

In this sense, the reduced use of opioids in our hospital is the expression of a cultural distortion on which unfortunately there is still a lot to work.

It is necessary to dwell on what could be solutions to the problems highlighted in this study.

We should certainly improve some aspects of pain assessment, but above all think of a different organization at least with regard to acute pain, and improve CME courses and information for parents and patients when possible. Facilitations for a correct pain management practice can be provided by a pain management service. At Ospedale Pediatrico Bambino Gesù IRCCS, pain management is currently ensured by ward doctors, assisted by a multidisciplinary commission for therapeutic standards and pain assessment and anesthesiologists are called only for the most difficult cases. Unfortunately this reality is widespread in much of the Italian territory, as demonstrated by Vittori et al. [38].

The comparison of the results from the two departments showed that this model did not facilitate overcoming the knowledge gap. This is more evident in the DS than in the DO where, in consideration of the fact that patients have recently undergone surgery and that therefore a higher intensity of pain is expected, it would be necessary to change some aspects: (1) Establish an Acute Pain Therapy Service with dedicated staff and (2) bring the pain assessment to every 6 h instead of the current one every 8 h. The implementation of an acute pain service improve the postoperative care of children and adults, and since 2010 the Royal College of Anesthetists in the United Kingdom suggested that a member of the Acute Pain Service visit all the children undergoing major surgery and a nurse from the pain service visit the wards each day in order to support nurses giving care for children in pain [39, 40]. This could have facilitated the decision-making process for children in pain and could have increased nurses’ confidence regarding pain management [41].

For all hospital health workers, in addition to the commitment to continuing education, it would be necessary to focus attention also on effective and safe use of drugs, especially opioids, alongside an intensification of psychological therapy, non-drug therapy and set up courses for parents and children [42, 43].

## Conclusion

Although the sample collected was lower than expected, the results obtained can still show where we come from and where we are going. The suffering of young patients constantly requires a special daily attention from health professionals aimed at mitigating the components of intractable pain and resolving those of treatable pain through an iron organization to anticipate, detect, and mitigate patients’ distress and to measure and implement strategies that prevent the dysfunction that causes pain.

## Electronic supplementary material

Below is the link to the electronic supplementary material.

Supplementary Material 1

## Data Availability

The datasets used and/or analysed during the current study are available from Giuliano Marchetti on reasonable request.

## Abbreviations

AS: Analgesic Score
CME: continuing medical education
DO: Department of Onco-Hematology and Cell and Genetic Therapy
DS: Department of Surgery
FLACC: Face, Legs, Arms, Cry and Consolability
M/SP: moderate /severe pain
NRS: Numerical Rating Scale
PMI: Pain Management Index
VAS: Visual Analogic Scale
WHO: World Health Organization
