# Supplementary material for: Pain prevalence and pain management in children and adolescents in an italian third level pediatric hospital: a cross-sectional study
Source: Ital J Pediatr. 2023 Mar 29;49:41. doi: 10.1186/s13052-023-01439-2 (PMC10053721; doi:10.1186/s13052-023-01439-2)
Supplement: Supplementary file 1 — Supplementary Material 1 [file 13052_2023_1439_MOESM1_ESM.docx]

Dear Editor,

We thank you for the enormous work you have done by choosing highly experienced reviewers. We have followed all their suggestions in the revision of our manuscript, which we are now resubmitting. We also arranged for the manuscript to be edited by a native English speaker, as requested.

We are confident that it will be accepted and published in the prestigious and authoritative Journal you represent.

Kind Regards

Alessandro Vittori
